# Suitability of Marine- and Porcine-Derived Collagen Type I Hydrogels for Bioprinting and Tissue Engineering Scaffolds

**DOI:** 10.3390/md20060366

**Published:** 2022-05-30

**Authors:** Malachy Maher, Veronica Glattauer, Carmine Onofrillo, Serena Duchi, Zhilian Yue, Timothy C. Hughes, John A. M. Ramshaw, Gordon G. Wallace

**Affiliations:** 1 Intelligent Polymer Research Institute, ARC Centre of Excellence for Electromaterials Science, AIIM Facility, Innovation Campus, University of Wollongong, Wollongong, NSW 2519, Australia; mkm290@uowmail.edu.au (M.M.); carmine.onofrillo@unimelb.edu.au (C.O.); serena.duchi@unimelb.edu.au (S.D.); zyue@uow.edu.au (Z.Y.); 2 Commonwealth Scientific and Industrial Research Organisation, CSIRO Manufacturing, Clayton, VIC 3168, Australia; veronica.glattauer@csiro.au (V.G.); timothy.hughes@csiro.au (T.C.H.); 3 The Aikenhead Centre for Medical Discovery (ACMD), St. Vincent’s Hospital Melbourne, Fitzroy, Melbourne, VIC 3065, Australia; 4 Department of Surgery, St. Vincent’s Hospital, University of Melbourne, Parkville, VIC 3065, Australia; johnamramshaw@gmail.com

**Keywords:** collagen, hydrogel, stability, methacrylation, source, bioink, 3D printing

## Abstract

Collagens from a wide array of animals have been explored for use in tissue engineering in an effort to replicate the native extracellular environment of the body. Marine-derived biomaterials offer promise over their conventional mammalian counterparts due to lower risk of disease transfer as well as being compatible with more religious and ethical groups within society. Here, collagen type I derived from a marine source (*Macruronus novaezelandiae*, Blue Grenadier) is compared with the more established porcine collagen type I and its potential in tissue engineering examined. Both collagens were methacrylated, to allow for UV crosslinking during extrusion 3D printing. The materials were shown to be highly cytocompatible with L929 fibroblasts. The mechanical properties of the marine-derived collagen were generally lower than those of the porcine-derived collagen; however, the Young’s modulus for both collagens was shown to be tunable over a wide range. The marine-derived collagen was seen to be a potential biomaterial in tissue engineering; however, this may be limited due to its lower thermal stability at which point it degrades to gelatin.

## 1. Introduction

Collagen is a principal protein component in all animal species. The collagen family in humans and most higher vertebrates consists of 28 distinct types [1], of which type I collagen, the main component of skin, tendon, ligaments and bone, is the most abundant. Many of the other collagens are only minor components of the extracellular matrix (ECM), but have specific roles in particular tissue locations [1]. 

For decades, mammalian collagen has been extracted from by-products of the bovine and porcine processing industry. However, religious, personal, dietary and regulatory concerns surround the use of such sources. For example, transmissible diseases such as bovine spongiform encephalopathy (BSE), transmissible spongiform encephalopathy (TSE) and foot-and-mouth disease (FMD) are causes for concern, as well as religious constraints to Muslims, Hindus and Jews, who account for 38.4% of the global population [2]. These issues demonstrate a need for an alternative source of collagen and biomaterials. Marine-derived biomaterials including chitin and alginate have demonstrated the benefits of marine biomaterials [3,4], and more recently collagens from marine sources have attracted great interest as biomaterials due to their biocompatibility, high biodegradability, low immunogenicity and ease of extraction [5,6,7,8,9,10]. 

All collagens share a common structural motif, the triple-helix, in which three individual chains, each in a left-handed polyproline-II-like helix, are wound together to form a right-handed, rope-like triple helix [11]. This triple-helical structural motif leads to constraints on the amino acid sequence, with collagens being characterised by a repeating (Gly-Xaa-Yaa)_n_ sequence, as only Glycie (Gly) is small enough to fit within the triple-helical conformation [11]. Collagens are subject to a range of secondary modification reactions during biosynthesis [12]. Of these, the hydroxylation of Proline (Pro) residues in the Yaa position to give 4-hydroxyproline (Hyp), is the most frequent [13], and contributes significantly to the stability of collagens [14]. The degree of hydroxylation is related to the environmental temperature for a species [15]. Thus, warm-blooded animals, such as the pig, have high levels of hydroxylation, around 10% Hyp residues per type I collagen trimeric molecule, to provide collagen that is stable at a body temperature of around 37 °C. On the other hand, cold-blooded animals, such as most fish, have lower extents of hydroxylation commensurate with the required environmental stability. Hence, a tropical fish, such as Tilapia (*Oreochromis niloticas*), has around 8.5% Hyp, giving a melting temperature (T_m_) of 34 °C [16] while, at the other extreme, an Antarctic Ice-fish (*Trematomus eulepidotus*), has around 4.5% Hyp and a T_m_ of just 6 °C [17]. On the other hand, although fish collagens in solution are less stable with lower T_m_ values than mammalian collagens, they have the advantage of being less likely to introduce diseases when used in clinical settings [18]. However, the disadvantage of a lower T_m_ can be overcome by crosslinking, as this process typically gives a structure with a denaturation temperature than is approximately 20 °C greater than the T_m_ value [19].

Collagen, and its denaturation product, gelatin, have been used extensively as templates (scaffolds) for tissue engineering and regenerative medicine as they can form highly hydrated networks, termed hydrogels [20,21]. The various highly specific native interactions of collagen [22], which are frequently lost with denaturation, make collagen particularly suitable for creating a 3D environment that can support cell proliferation and differentiation [23,24]. An emerging interest is the opportunity to use collagen in bioprinting these templates. However, this presents challenges, as additional stabilisation is required to create mechanically viable 3D collagen templates, which, should not be detrimental to the associated cells.

Although a broad range of potential crosslinking approaches have been described [12], few are suitable for crosslinking of printed hydrogels as they are cytotoxic or are not suited for rapid reaction immediately following printing [25]. The use of methacrylated collagen [26] is gaining attention, especially following the many studies with methacrylated gelatin [27,28]. The methacrylated collagen can be rapidly cross-linked by exposure to UV irradiation with a photoinitiator present.

In the present paper, a marine collagen of the fish origin (MC) has been examined with respect to suitability for 3D printing. The MC has been compared with porcine collagen (PC), through examination of stability, before and after methacrylation, and the rheology, crosslinking kinetics and printing characteristics of the methacrylated collagens.

## 2. Results

Samples of both MC and PC were readily extracted and methacrylated. The extent of methacrylation was similar for both MC and PC samples with 51% (±6%, n = 3) and 58% (±4%, n = 3), respectively, of the Lys residues present in the sequence being modified. These values contrast those obtained for GelMA, which had also been modified using the same excess of methacrylic anhydride, to obtain 77% (±3%, n = 3) modified [29,30].

**Figure 1:**
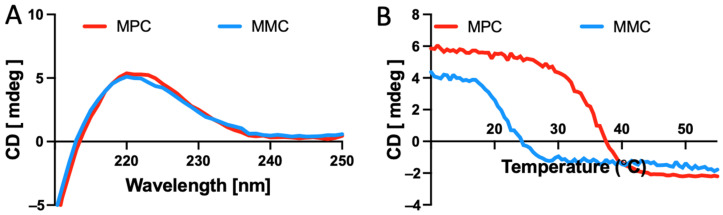
CD spectra of MMC and MPC. (**A**) Spectra scan showing absorption at 222 nm, demonstrating retention of the triple helix. (**B**) A temperature scan of the methacrylated collagens, at 222 nm, shows the MPC collagen has a relative thermal stability higher than the MMC.

Following methacrylation of the MC and PC, the triple helix remained intact and stable, with circular dichroism (CD) spectroscopy absorption peaking at 222 nm, as seen in Figure 1A, which is consistent with un-modified native collagens.

The thermal response curves, measured at 222 nm (Figure 1B), indicated that the methacrylated marine collagen (MMC) had significantly lower thermal stability than the methacrylated porcine collagen (MPC), as it denatured at approximately 12 °C lower. As the samples were heated, the triple helical structure of the collagen was seen to lose its tertiary structure and degrade to gelatin.

Hydrogels were prepared by dissolving MMC and MPC at 30 mg/mL in 20 mM acetic acid, at 4 °C. Immediately prior to use, the sample was neutralised with 1 M NaOH to a pH of 7.5–8.5, and with 10× PBS to achieve a 1× concentration. LAP was added at 0.05%.

**Figure 2:**
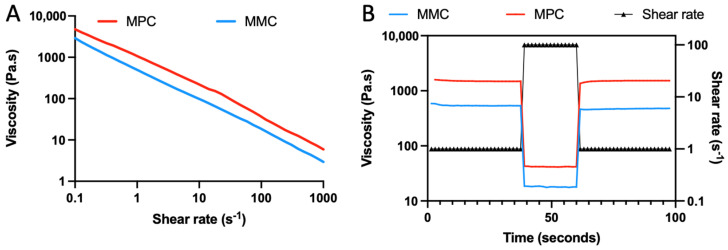
Rheological scans of MMC and MPC samples. (**A**) Shear rate ramp demonstrates that both MMC and MPC display shear thinning throughout the 0.1–1000 s^−1^ range. (**B**) Shear events alternating between 0.1 and 100 s^−1^ show the collagen samples both exhibit rapid thixotropy and viscosity recovery.

Rheological data (Figure 2) indicated that both MMC and MPC samples exhibited shear thinning in response to applied shear, making them suitable for extrusion bioprinting. The viscosity of both samples (~100 Pa.s at a shear rate of 10 s^−1^), when prepared at 3% *w*/*v* was also suitable for extrusion printing, matching established bioinks [31]. The samples were seen to have rapid recovery following shear events, displaying thixotropic behavior, recovering and increasing in viscosity 2-magnitudes within 2 s of a exiting a high-shear environment (100 s^−1^).

**Figure 3:**
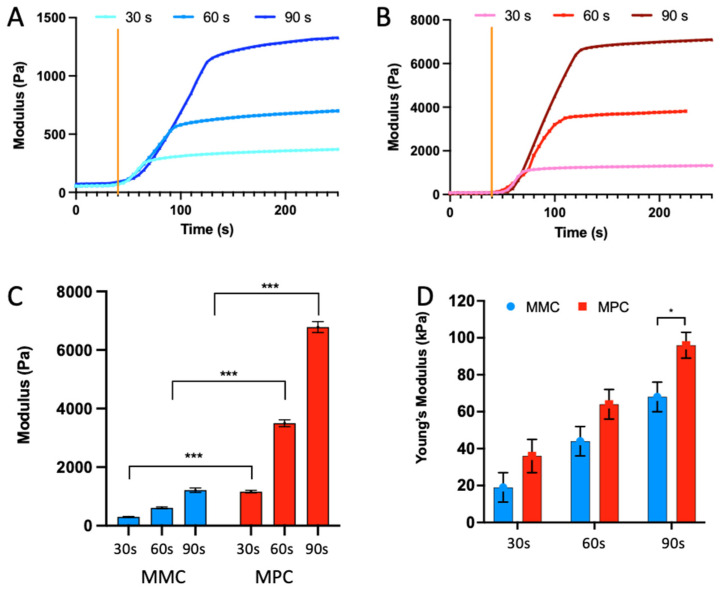
Mechanical properties of crosslinked collagens. (**A**,**B**) Crosslinking kinetics of the MMC and MPC in response to UV light exposure (400 nm and 20 mW/cm^2^) for 30, 60 and 90 s. The orange bar in (**A**,**B**) represents the point at which the UV light source was initiated. (**C**) Peak moduli observed by the rheometer following the photo-crosslinking. (**D**) Young’s Modulus of the crosslinked collagen templates during unconfined compression to 20% strain. The linear region of the compression response was used to calculate the Young’s Modulus. Results from the *t*-test indicate significant differences between the modulus of MMC and MPC following exposure to 30, 60 and 90 s of 400 nm light (*** *p* < 0.001), with MPC resulting in higher moduli. The *t*-test following compression tests, shows the Young’s Modulus of the MPC was significantly higher than the MMC following 90 s of 400 nm light exposure (* *p* < 0.05).

When exposed to UV light (400 nm, 20 mW/cm^2^), rheological data shown in Figure 3 show both collagens exhibited rapid crosslinking, with templates solidifying to remain intact in under 30 s. The MMC underwent crosslinking, increasing in modulus at a rate of ~600 Pa/min, whereas the MPC increased at ~3500 Pa/min. Increased exposure time saw an increase in the modulus achieved, with MMC achieving peak moduli of 34 ± 19, 612 ± 29 and 1214 ± 74 Pa after exposure for 30, 60 and 90 s, respectively. Comparatively, MPC achieved peak moduli of 1164 ± 43, 3519 ± 113 and 6784 ± 184 Pa, respectively (n = 3).

These stark differences were less pronounced in compression testing. The MMC and MPC samples were cast in moulds (2 mm ø, 2 mm height) by exposure to UV light for 30, 60 and 90 s. These templates underwent unconfined compression and the MMC templates reported Young’s Moduli of 19 ± 8, 44 ± 7 and 68 ± 8, at the increasing UV exposure times, and the MPC samples 36 ± 9, 64 ± 8 and 96 ± 7, respectively (n = 3). A significant difference was observed between the MMC and MPC with 90 s of exposure.

**Figure 4:**
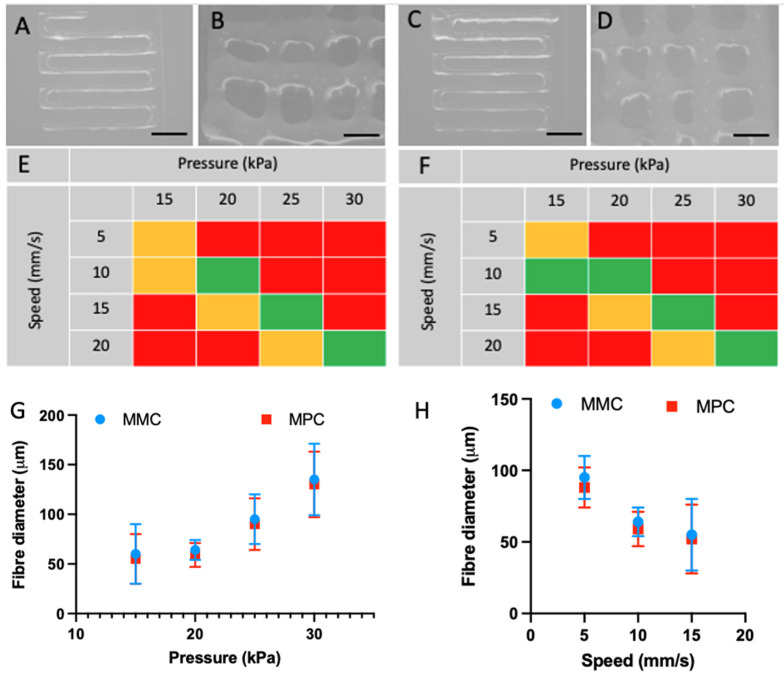
3D extrusion printing of collagen hydrogels. (**A**,**B**) MPC 3D printed scaffolds, at 1 and 2 layers (scale bars are 200 and 100 µm, respectively). (**C**,**D**) MMC 3D printed scaffolds, at 1 and 2 layers (scale bars are 200 and 100 µm, respectively). (**E**,**F**) Schematic showing the conditions that MPC and MMC are able to be extrusion printed with a 22 G nozzle. The red zones represent conditions that are not suitable for 3D extrusion printing, the yellow show possible conditions, and the green zones show ideal conditions (**G**,**H**) show the fibre diameter achieved at varying speeds (pressure fixed at 20 kPa) and variable pressure (speed fixed at 10 mm/s).

The MMC and MPC hydrogels were successfully extrusion 3D printed. At a fixed pressure of 20 kPa and speed of 10 mm/s, both hydrogels were able to be extruded with consistent, continuous flow. The MMC had a slightly lower viscosity, and as such was able to be extruded at slightly lower pressures, as seen in Figure 4. The fibre diameters were obtained using MMC and MPC were comparable, with no significant differences seen (averages of 64 µm for MMC, and 59 µm for MPC, n = 5). Many recent studies that utilise collagen in extrusion printing rely on a composite material in order to optimise the materials’ rheological properties for the printing process, and the subsequent biological interaction. In such examples we have seen collagen blended with calcium phosphate for orthopaedic applications [32] as well as fibrin [33] and alginate [34] amongst others for a wide range of applications. While many 3D printing and crosslinking techniques have been explored [35,36], including drop-on-demand studies [37], as this study relied upon a pure collagen solution, the methacrylation and photo-crosslinking enabled structures to be crosslinked.

**Figure 5:**
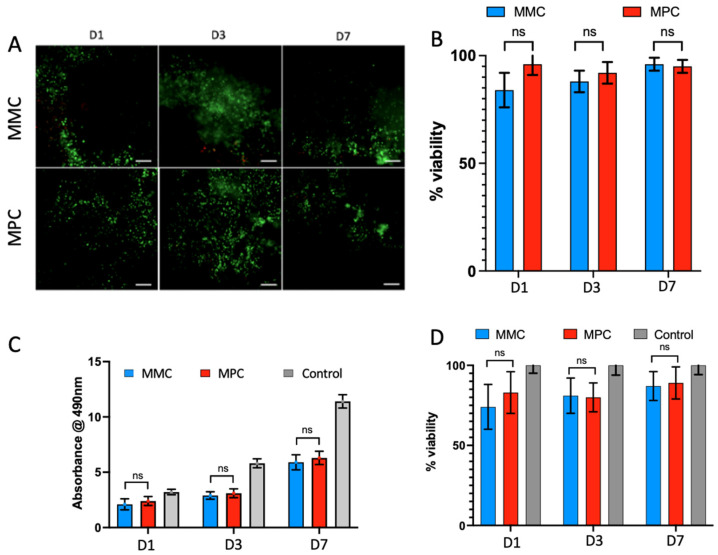
Cell viability of encapsulated L929 fibroblasts in crosslinked MMC and MPC collagen hydrogels. (**A**) Representative images of bioscaffolds stained with live/dead (calcein AM, ethidium homodimer-1) at the 3 different indicated time points (D1, 3 and 7). Scale bar is 100 µm. (**B**) The graph shows the cell viability obtained from the live/dead stained samples, calculated as the percentage of live: dead cells. (**C**) The graph shows the metabolic activity of the cells in the bioprinted scaffolds evaluated with MTS assay and absorbance readings at 490 nm. (**D**) Cell viability calculated from MTS assay, normalised to standard curve of 2D cultured cells. Results of the *t*-test indicate no significant difference between MMC and MPC at each timepoint in both the calcein-AM and ethidium homodimer-1 staining, as well as the MTS assay. ns: No statistical significance was observed between MMC and MPC at each timepoint.

L929 fibroblasts were successfully encapsulated in MMC and MPC based hydrogels, with live/dead stain (calcein AM, ethidium homodimer-1) confirming viability in the MMC templates of 84 ± 8, 88 ± 5 and 95 ± 4% at days 1, 3 and 7 (n = 3), respectively. Crosslinked MPC saw cell viability of 94 ± 5, 92 ± 4 and 94 ± 3%, respectively (n = 3). No significant differences were noted between the materials at any time point, as seen in Figure 5. Results of the MTS metabolic assay, compared to the standard curve generated from traditional 2D cell culture of an increasing numbers of cells, saw relative cell viability of 74 ± 14, 81 ± 11 and 87 ± 9% in the marine methacrylate samples at day 1, 3 and 7, while cells encapsulated in the MPC group recorded relative cell viability of 83 ± 13, 80 ± 9 and 89 ± 10% (n = 3). No significant differences were observed between groups at any timepoint.

## 3. Discussion

While several studies have demonstrated that the amino acid sequences of marine-derived collagen are similar to those observed with mammalian-derived collagens, several key differences do exist. Marine-derived collagens exhibit a high degree of structural similarity between species with respect to individual α1 and α2 chains [38,39,40]. Furthermore, they exhibit a lower content of imino acids (Pro and Hyp), including a lower level of hydroxylation, and higher levels of serine and threonine residues, compared to mammalian-derived collagens [41,42]. These differences (particularly the decrease in imino acid content and hydroxylation) result in changes to physical properties, such as rheological properties and thermal stability [43,44,45]. When extracting MC, the type I collagen is the only collagen component, compared to porcine extraction which requires separation of type I from type III [46].

While both marine- and porcine-derived collagens were methacrylated under the same conditions, the degree of functionalisation (DOF) obtained for MC and PC (51 ± 6% and 58 ± 4%, respectively) was significantly different. This is compared to gelatin where in the DOF was found to be 77 ± 3% substitution, likely due to differences in pKa values for Lys residues in the unstructured gelatin compared to the triple-helical collagen, where steric hinderance can limit availability. Ultimately, the collagens underwent a sufficient level of methacrylation allowing effective photo-crosslinking.

Circular Dichroism adsorption as a function of temperature indicated that MMC denatured at lower temperate (25 °C) when compared with MPC (37 °C). This difference is largely due to fact that the MC contains less imino acids (hydroxyproline) than the MPC [47]. The hydroxyproline content of fish species is associated with the water temperature of their normal habitat, with cold water fish (including Blue Grenadier) reporting a hydroxyproline content as 35–37% of the Proline residues modified, compared to warmer water fish species, such as tilapia, where the extent of methacrylation is 43% [48,49]. As the denaturation temperature of the MC is less than human body temperature, the MC constructs require additional stabilisation to be used in vivo for biomedical applications. Interestingly, Ehrlich et al. found that hydroxylated collagens (Hyalonema sieboldi; Porfifera, Class Hexactinellidia) form a template that is primed for mineralisation [50] Crosslinking is frequently used for mammalian collagens, as this enables control and lengthening of the turnover of implanted material. While various crosslinking techniques have been employed to stabilise collagen, methacrylation is becoming increasingly common. Once exposed to UV-irradiation, the network is stabilised at higher temperatures, that enable use as an implant at body temperature. In the present study, on bioprinting, stabilisation is necessary and already incorporated aspect of the printing process, so stability is no longer an issue.

To be used as a bioink in 3D printing, specific rheological properties are required, including appropriate viscosity and shear thinning with rapid recovery following extrusion [31]. When prepared at 3% *w*/*v*, the collagen solutions exhibited a viscosity and shear thinning profile that is suitable for bioprinting [51]. Both collagens underwent shear thinning, which results in less pressure being needed for printing, and particularly a reduction in stress that cells experience as they are extruded through the 3D printer nozzle (Figure 2). The MMC displayed a slightly lower viscosity compared to the MPC, a phenomenon which has been reported in the literature and attributed to varying amino acid sequences [44]. This lowered viscosity likely translates to decreased stress on cells during the extrusion process. Following the extrusion process, as the bioink is deposited from the nozzle, it needs to rapidly recover and increase in viscosity to support itself, in a process termed thixotropy [51]. Following a simulated high stress—relaxation environment using the rheometer, the marine and MPC underwent rapid thixotropy, recovering two magnitudes in viscosity near instantaneously (within 2 s). This rapid recovery enables high shape fidelity in printed templates, which allows higher resolutions to be achieved.

Once extruded, exposure to UV-irradiation is used to crosslink the templates and stabilise the collagen. When exposed to 400 nm irradiation, both samples underwent rapid crosslinking, increasing in modulus at a rate of ~600 Pa/minute and ~3500 Pa/minute for the MMC and MPC, respectively (Figure 3). Exposure for 30, 60 and 90 s saw a uniform rate of crosslinking throughout the exposure timeframe, with the MMC achieving peak moduli of 19 ± 34, 612 ± 29 and 1214 ± 74 Pa, respectively. Comparatively, MPC achieved peak moduli of 1164 ± 43, 3519 ± 113 and 6784 ± 184 Pa, respectively, a significant increase at each time point (*p* < 0.05) compared to the MMC. These rapid rates of crosslinking were critical to achieving 3D printed templates of high resolution. The stark difference in the moduli achieved between the samples could be due to the small difference in degree of methacrylation, or due to differences in amino acid sequence which effect the degree of hydration and physical measures such as viscosity. Interestingly, these differences were less pronounced when looking at the Young’s modulus of the samples during compression, with crosslinked MMC reporting Young’s modulus of 19 ± 8, 44 7 and 68 ± 8 kPa, at the increasing UV exposure times, and the MPC samples 36 ± 9, 64 ± 8 and 96 ± 7 kPa, respectively. The ability to tailor the modulus of the hydrogel scaffold is favourable for tissue engineering, as it allows the environment to be tailored for specific applications. For example, templates designed for soft tissue regeneration, including neural regeneration, prefer a soft environment of 0.1–1 kPa, whereas muscle ~ 10 kPa and bone ~ 30 kPa prefer stiffer materials [52,53].

Both the MMC and MPC were extrusion-3D-printed. The resolution achievable is consistent with other examples of mammalian-based collagen hydrogel extrusion, as well as other collagen–pluronic blends [54,55]. While the MMC exhibited slightly lower viscosity than the MPC, this did not translate to any significant difference in fibre diameter.

L929 fibroblasts were encapsulated in the collagen hydrogels and crosslinked for 60 s via exposure to 400 nm light at 20 mW/cm^2^. Cell viability was measured at days 1, 3 and 7 via live/dead stain and confirmed above 80% at all timepoints, whereas an MTS assay confirmed this result with cell viability typically above 80% compared to a traditional 2D cell culture standard curve. There was a small decrease in cell viability at day 1, particularly in the MMC; however, this is likely due to the stress of cell encapsulation of casting protocol. Overall, the cells displayed high cell viability, indicating the collagens cytocompatibility. The minor decreases in cell viability in the encapsulated MMC and MPC are likely attributable to the free radicals produced during crosslinking, and accurate control over the pH and ionic strength is critical to ensuring high cell viability, as achieved in Figure 5.

A different study examined the biocompatibility of Saos-2 cells when encapsulated in a methacrylated collagen hydrogel. This study found differences based on the photo-initiator that was used with VA086 giving better cell viability than Irgacure 2959 (I2959) [56]. In the present study, the use of LAP as a photo-initiator was preferred compared to photoinitiators that are excitable at lower wavelengths, as it can be excitated at higher and less damaging wavelengths of 400 nm and was found to give high cell viability.

Marine collagens have been utilised in combination with cells and demonstrated not only high cell viability, but also upregulation of key differentiation markers [57]. A biphasic salmon and jellyfish collagen scaffold was examined for treatment of osteochondral defects. This scaffold supported both osteogenic and chondrogenic differentiation of bone marrow-derived hMSC’s, highlighting the potential of MC to act as an osteochondral scaffold [58].

## 4. Materials and Methods

### 4.1. Collagen Samples

Collagen type I was extracted from the skin of blue grenadier fish (*Macruronus novazealandii*) [47], and from solvent extracted (dichloromethane) porcine skin. In both cases, the skin was then cut into small sections (~10 × 10 mm) and suspended at ~5 mg/mL in 50 mM acetic acid (Chem Supply, Port Adelaide, South Australia, Australia) adjusted to pH 2.5 with HCl (Sigma Aldrich, St. Louis, MO, USA), at 4 °C for 16 h. Pepsin was added at 1 mg/mL. After digestion, samples were clarified by centrifugation at 7500× *g* for 120 min at 4 °C. Collagen was precipitated by addition of NaCl (Sigma Aldrich, St. Louis, MO, USA) solution to 0.7 M and then standing at 4 °C for 16 h. The precipitate was collected by centrifugation, 2500× *g* for 30 min, then exhaustively dialysed exhaustively against 20 mM acetic acid and freeze dried. The porcine collagen (PC) was further fractionated to remove type III collagen. It was resuspended in 0.2 M NaCl in 50 mM sodium phosphate buffer at pH 7.2. NaCl was then added to 1.7 M final concentration and then standing at 4 °C for 16 h. The precipitate was removed by centrifugation at 7500× *g* for 120 min at 4 °C. The supernatant was then taken to 2.4 M NaCl. After standing at 4 °C for 16 h, the precipitate of purified type I collagen was collected by centrifugation at 2500× *g* for 30 min, then dialysed exhaustively against 20 mM acetic acid and freeze-dried.

Methacrylated collagen was prepared by dissolving the collagen samples at 3 mg/mL in 20 mM acetic acid and then adjusted to 200 mM NaCl and taken to pH 7.5 with NaOH, all at 4 °C. A 5-fold molar excess of methacrylic anhydride was added and the pH maintained at 7.5 using NaOH for 4 h. The reaction was left overnight for completion and then samples were dialysed exhaustively against 20 mM acetic and freeze-dried. The extent of collagen modification was determined in triplicate using a 2,4,6-trinitrobenzene sulfonic acid (TNBSA) assay [59] (Thermo Scientific, Waltham, MA, USA) following the manufacturer’s instructions [47].

### 4.2. Circular Dichroism

Collagen samples were dissolved in H_2_O at 0.1 mg/mL at 4 °C. Circular dichroism (CD) spectra were collected using samples in 0.1 mm quartz cuvettes using a JASCO J-815 instrument (Jasco, Easton, MD, USA). Spectra were collected from 400–200 nm wavelength while at 4 °C, whereas temperature scans were performed at 222 nm at 0.3 °C/min from 10–55 °C.

### 4.3. Rheology

Collagen samples were prepared by dissolving at 30 mg/mL in 20 mM acetic acid at 4 °C. Rheology measurements were performed with an Physica MCR 301 Rheometer (Anton Paar, Graz, Austria) fitted with a Peltier temperature controller and connected to an EXFO Acticure 4000 light source. Measurements were made using a 15 mm 1° conical plate. Immediately prior to measuring, collagen samples were neutralised to pH of 7.25 ± 0.25 with 1 M NaOH, and the salt concentration balanced to 1× PBS using 10× PBS solution. Samples of 0.1 mL were used and added to the Peltier stage at 5 °C, and set at a gap distance of 500 µm. Samples were then held for a pre-shear period to remove prior memory, using a shear rate of 5 s^−1^ for 2 min at 4 °C. A shear rate ramp was performed from 0.1–1000 s^−1^ while at 5 °C. To examine viscosity recovery and thixotropy, a series of shear events were applied at 1000 s^−1^, before returning to a resting rate of 0.1 s^−1^, again at 5 °C. The photo crosslinking kinetics of the methacrylated collagens were examined by inclusion of lithium phenyl-2,4,6-trimethylbenzoylphosphinate (LAP) was dissolved in samples at 0.05% (Sigma Aldrich, St. Louis, MO, USA). These samples were monitored at a fixed frequency of 1 Hz and amplitude of 1%, for an initial 30 s, before being exposed to 405 nm light at 20 mW/cm^2^ for 60 s. The samples were monitored for an additional 5 min, at 5 °C.

### 4.4. Mechanical Testing

The photo-crosslinked hydrogels, prepared as above (Section 4.3) were also subject to mechanical testing using the TA Electroforce 5500 mechanical loading device (TA Instruments, New Castle, DE, USA) fitted with a 250 g load cell. Collagen templates were prepared by casting into moulds (2 mm ø, 2 mm height) and photo-crosslinked for 30, 60 and 90 s, respectively, by exposure to 400 nm light at an intensity of 20 mW/cm^2^ (OmniCure, Series 1500). The templates underwent 20% unconfined compression and the Young’s Modulus was calculated from the linear region of the response curve.

### 4.5. 3D Extrusion Printing

Marine- and porcine-derived collagen type I were prepared at 30 mg/mL in 20 mM acetic acid at 4 °C. Immediately prior to use, the solution was neutralised with 1 M NaOH, adjusted to 1× PBS with 10× PBS and the photo-initiator LAP added at 0.05% *w*/*v*. The GeSim Bioscaffolder 3.2 (GeSim, Radeberg, Germany) was used for printing, with a 22 G tip (Nordson EFD, East Providence, RI, USA). The cartridge was held at 5 °C and the plate was at 10 °C. A gap distance of 0.5 mm was used, and the print speed was fixed at 10 mm/s with the extrusion pressure held at 20 kPa. Samples were printed as single layers, and between successive layers, UV light (Detail wavelength) was applied to crosslink at every second layer, at an intensity of 20 mW/cm^2^ for 20 s.

### 4.6. Cell Viability

Again, marine- and porcine-derived collagen type I were prepared at 30 mg/mL in 20 mM acetic acid at 4 °C. Immediately prior to use, the solution was neutralised with 1 M NaOH, adjusted to 1× PBS with 10× PBS (Thermo Scientific, Waltham, MA, USA) and the photo-initiator LAP added at 0.05% *w*/*v*. L929 fibroblasts (Thermo Scientific, Waltham, MA, USA) were encapsulated at a density of 2 × 10^6^ cells/mL, and cast (2 mm ø, 2 mm height), with crosslinking completed using 400 nm light at an intensity of 20 mW/cm^2^ for 60 s. Cast scaffolds were placed in a 24 well plate and maintained with 2 mL of complete MEM culture media (Gibco, Waltham, MA, USA), with FBS (Gibco, Waltham, MA, USA), NEAA (Gibco, Waltham, MA, USA) and 1 × antibiotic-antimycotic (Gibco, Waltham, MA, USA). At days 1, 3 and 7, cell viability, proliferation and cytocompatibility were investigated via MTS assay (Abcam, Cambridge, UK) and live/dead assay (ethidium homodimer-1 and calcein-AM, Thermo Fischer, Waltham, MA, USA). The MTS assay was measured using plate reader at 490 nm, and compared with a standard curve generated from traditional 2D cell culture with known numbers of cells, and live/dead cells were imaged using Nikon Eclipse Ti microscope (Nikon, Tokyo, Japan) with a 10× objective lens.

## 5. Conclusions

This study demonstrates the potential of a MMC for use as a bioink, which could aid in regenerative medicine applications. Compared with the MPC, both materials retained structural stability during the extraction and methacrylation process, as well as favourable rheological properties. Importantly, extrusion printing was possible and L929 fibroblasts displayed high cell viability when encapsulated in both materials. However, key differences were seen, including the MMC having a lower denaturation temperature, but this is not of concern after the crosslinking needed to produce stable bioprinted scaffolds/templates. Nevertheless, care is needed to avoid unintentional denaturation during handling. Further advantages of the MC include that the lower shear stress during printing may assist in cell viability, and the crosslinking is rapid and effective, retaining cell viability. The MC is readily purified and does not need further fractionation like mammalian collagen to remove any type III collagen that is present. A low type III content is preferable as it is considerably more immunogenic than type I collagen [60]. Furthermore, there is a lower risk of disease transfer when using MC and it is acceptable to a higher proportion of the population where personal, ethical or religious grounds preclude porcine or bovine materials. The present study has used a moderately stable MC, but it is probable that for biomedical applications one of the MC sources that is more thermally stable could be used. Future studies should be targeted to investigate the biological potential of cell laden marine collagen hydrogel scaffolds, which allow for a safe biomaterial that is accessible to a greater portion of the population than current mammalian-derived collagen sources.
